# Anterior debridement combined with autogenous iliac bone graft fusion for the treatment of lower cervical tuberculosis: a multicenter retrospective study

**DOI:** 10.1186/s10195-023-00730-6

**Published:** 2023-09-14

**Authors:** Ping Xia, Pengfei Tao, Xiaolong Zhao, Xianglin Peng, Songfeng Chen, Xiucai Ma, Lei Fan, Jing Feng, Feifei Pu

**Affiliations:** 1 Department of Orthopaedics, Wuhan Fourth Hospital (Puai Hospital), Wuhan, China; 2 Department of Orthopedics, Traditional Chinese and Western Medicine Hospital of Wuhan (Wuhan No.1 Hospital), Tongji Medical College, Huazhong University of Science and Technology, Wuhan, China; 3 Department of Orthopaedic Surgery, The First Affiliated Hospital, Zhengzhou University, Zhengzhou, China; 4 Department of Bone and Soft Tissue Oncology, Gansu Provincial People’s Hospital, Lanzhou, China; 5 Department of Orthopedics, The Second Affiliated Hospital of Nanjing Medical University, Nanjing, China

**Keywords:** Cervical tuberculosis, Bone fusion, Bone graft, Lesion removal

## Abstract

**Background:**

This study aimed to analyze the clinical efficacy of one-stage anterior debridement of lower cervical tuberculosis using iliac crest bone graft fusion and internal fixation.

**Materials and methods:**

A retrospective analysis was performed on 48 patients with lower cervical tuberculosis admitted to multiple medical centers from June 2018 to June 2021. Among them, 36 patients had lesions involving two vertebrae and 12 patients had lesions involving more than three vertebrae. All patients were treated with quadruple antituberculosis drugs for more than 2 weeks before the operation, and then treated with one-stage anterior debridement and autogenous iliac bone graft fusion combined with titanium plate internal fixation. After the operation, antituberculosis drugs were continued for 12–18 months. The patients were followed-up to observe the improvement in clinical symptoms, bone graft fusion, Cobb angle, visual analog score (VAS), erythrocyte sedimentation rate (ESR), C-reactive protein (CRP), wound healing, and neurological function.

**Results:**

The patients were followed-up for 13–43 months, with an average of 21.46 ± 1.52 months. The clinical symptoms significantly improved after the operation. The bone graft was completely fused in all patients, and the bone fusion time was 3–6 months, with an average of 4.16 ± 0.47 months. At the last follow-up, the Cobb angle, VAS, ESR, and CRP level were significantly lower than those before surgery (*P* < 0.05). None of the patients had loosening, detachment, or rupture of the internal fixation, and no recurrence occurred. All surgical incisions healed in one stage without infection or sinus formation. The preoperative Frankel neurological function classification was grade B in 7 cases, grade C in 13, grade D in 18, and grade E in 10. At the last follow-up, 8 cases recovered to grade D and 40 recovered to grade E.

**Conclusions:**

For patients with lower cervical tuberculosis, based on oral treatment with quadruple antituberculosis drugs, direct decompression through anterior debridement, followed by autologous iliac bone graft fusion combined with internal fixation can completely remove tuberculosis foci, rebuild the stability of the cervical spine, and obtain good clinical efficacy.

*Level of evidence* Level 3.

## Background

Tuberculosis is a relatively old disease; the development of antituberculosis chemotherapy drugs has greatly improved the prognosis and epidemic trend of tuberculosis [1]. Spinal tuberculosis is one of the most common types of bone and joint tuberculosis. In recent years, the incidence of spinal tuberculosis has been rising annually, and drug resistance in tuberculosis has become increasingly serious. The disease often develops rapidly, and spinal structural destruction appears quickly [2].

Although cervical tuberculosis is relatively rare, the incidence of paraplegia is high because of the presence of the cervical spinal cord in the spinal canal [3]. Chemotherapy is still the main treatment for cervical tuberculosis; however, with the wide application of internal fixation technology and the improvement of implant materials, surgical treatment of cervical tuberculosis has made rapid progress [4]. Because cervical tuberculosis often destroys the anterior column of the vertebral body, which can lead to vertebral instability, vertebral wedge changes, and kyphosis, anterior debridement combined with bone graft fusion and internal fixation has become a common surgical method for the treatment of cervical tuberculosis, with high safety and reliability [5].

Anterior intervertebral structural bracing and bone grafting can not only fully restore the height and physiological curvature of the vertebral body but also immediately restore the mechanical conductivity of the cervical spine and reconstruct the stability of the spine [6]. Autologous bone has good biocompatibility, conductivity, and inducibility, and is the first choice for bone transplantation. Commonly used autologous bones include the iliac crest, ribs, and peroneal bone [7].

Therefore, this study retrospectively analyzed the clinical data of 48 patients with lower cervical tuberculosis who were treated with anterior debridement combined with iliac crest allograft fusion and internal fixation from June 2018 to June 2021 in multiple medical centers to explore a more reasonable alternative material for cervical tuberculosis bone graft.

## Materials and methods

### Inclusion criteria

The inclusion criteria were as follows:

(i) The diagnosis of cervical tuberculosis was confirmed based on typical clinical manifestations, imaging examination, laboratory examination, postoperative histological examination, and pus culture.
(ii) The cervical spine was mainly damaged by the anterior column, with obvious spinal instability kyphosis or abscess formation in the spinal canal, with obvious neck pain and neurological symptoms.
(iii) Patients were treated with quadruple antituberculous drugs for 2–4 weeks, and erythrocyte sedimentation rate (ESR), C-reactive protein (CRP) level, and general condition improved.
(iv) According to the Xiangya Institutes of Medical Sciences cervical tuberculosis grading system [8], all patients were in grade 2, and the patients underwent anterior debridement combined with autogenous iliac bone graft fusion and plate fixation.
(v) The lesions did not involve the upper cervical vertebrae (atlas and axis) or thoracic vertebrae.

### Exclusion criteria

The exclusion criteria for both groups were as follows:

(i) Patients with active pulmonary tuberculosis.
(ii) Patients with nonspecific spinal infections or suspected other specific infections such as Brucella spondylitis.
(iii) Patients with severe cardiopulmonary dysfunction who cannot tolerate surgery and anesthesia.
(iv) Patients who cannot receive iliac crest graft from the body.
(v) Patients who were resistant to antituberculosis therapy.
(vi) Patients with upper cervical or skipping multilevel spinal tuberculosis.

### Patients

A total of 48 patients with lower cervical tuberculosis who were admitted to multiple clinical treatment centers from June 2018 to June 2021 with complete follow-up data and met the inclusion and exclusion criteria were selected. The study was reviewed and approved by the Ethics Committee of Traditional Chinese and Western Medicine Hospital of Wuhan (Wuhan No. 1 Hospital), Tongji Medical College, Huazhong University of Science and Technology. All patients provided written informed consent.

### Preoperative examination

Routine blood tests, ESR, CRP level, liver and kidney function, and tuberculosis antibody levels were measured preoperatively. Imaging examinations included cervical spine X-ray, computerized tomography (CT) and magnetic resonance imaging (MRI), all of which revealed vertebral bone destruction, vertebral collapse, vertebral space stenosis or disappearance, cervical canal stenosis of different degrees, paravertebral abscess, cervical kyphosis, and other imaging manifestations.

### Preoperative preparation

All patients were given continuous neck brace immobilization, bed rest, and nutritional support immediately after admission. Before surgery, the patients were treated with isoniazid (300 mg daily), rifampicin (450 mg daily), pyrazinamide (1500 mg daily), and ethambutol (750 mg daily) for more than 2 weeks. The patient’s ESR decreased, the symptoms of tuberculosis poisoning were alleviated, and the systemic symptoms improved. An anterior longitudinal incision of the sternocleidomastoid muscle was used for lesions involving three vertebral bodies, and a transverse incision of the right anterior neck was used for the rest.

### Procedure of operation

**Fig. 1:**
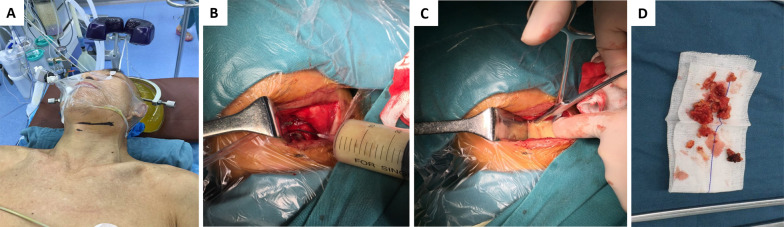
The process of surgical manipulation. **A** The surgical incision of the patient. **B**–**D** Anterior surgery was performed to completely remove pus, dead bone, and necrotic disc

After general anesthesia, the patient was placed in a supine position with the shoulder and neck pads high, and the cervical spine was kept in a slightly posterior extension position. The routine operation area was sterilized and covered with towels. A transverse incision was made on the right anterior side of the neck (Fig. 1A). The abscess was dissected and separated layer by layer to completely remove the prevertebral abscess. Under fluoroscopy using a C-arm X-ray machine, the diseased cervical disc was located and the anterior part of the diseased cervical spine was fully exposed. A bracing nail was fixed on the anterior side of the upper and lower vertebrae of the diseased segment, and the space between the diseased vertebrae was opened using a bracing device. The diseased disc, dead bone, and hyperplastic granulation tissue were completely removed, spinal cord compression of the neck was relieved, and the endplates of the adjacent upper and lower vertebral bodies of the diseased segment were scratched (Fig. 1B–D). A three-sided cortical bone was obtained from the anterior superior iliac spine, trimmed, and implanted between the upper and lower vertebrae adjacent to the resected lesion vertebrae, and an anterior cervical locking plate was placed. Complete hemostasis, suturing, and other operations were then performed.

### Postoperative treatment

Routine postoperative analgesia, dehydration, nutritional nerve, intravenous nutritional support, antituberculosis drugs and other treatments, blood transfusion, and albumin supplementation were administered when necessary. The incision and drainage were observed, and the drainage tube was removed when the drainage volume was less than 10 mL/24 h after the operation and ipratropium bromide was administered via inhalation for 5–7 days. A neck brace was worn for 3–4 months immediately after surgery. Isoniazid, rifampicin, pyrazinamide, and ethambutol quadruple antituberculosis drugs were continued postoperatively, and hepatoprotective therapy was administered for 3–6 months. They were then treated with antituberculous drugs, including isoniazid, rifampicin, ethambutol, and hepatoprotective therapy for 12–18 months. Liver and kidney function, blood routine, ESR, and CRP level were reviewed every month after discharge and then changed every 3 months after the patient was relatively normal. Follow-up was performed at 3, 6, 12, 18, and 24 months postoperatively.

### Efficacy evaluation

The perioperative indicators of the patients were recorded in detail, and the improvement of clinical symptoms, healing of the bone graft after surgery, and pain in the iliac crest area were evaluated. The local sagittal Cobb angle of the cervical spine, visual analog score (VAS) of the cervical spine, ESR, CRP level, and Frankel classification of neurological function were compared before surgery and at the last follow-up after surgery.

### Statistical analysis

The patient’s pain level and limb function were analyzed using SPSS 20.0 (IBM, USA). All indicators before and after surgery were expressed as x̅ ± *s* and compared using a paired *t*-test. Statistical significance was set at *P* < 0.05.

## Results

### Basic information

Among the 48 patients, 28 were male and 20 were female. The average age was 58.3 ± 5.2 years, ranging from 27 to 71 years. The disease duration was 4–13 months, with an average of 5.9 ± 2.7 months. All patients were diagnosed with lower cervical tuberculosis based on the postoperative pathology.

All patients had different degrees of tuberculosis poisoning symptoms, such as low fever, weight loss, night sweats, fatigue, emaciation, persistent neck pain, limited activity, and other symptoms. Among them, 38 patients had different degrees of symptoms of cervical spinal nerve function injury, including limb numbness, unstable holding, walking on cotton, walking instability, and incomplete paralysis. The preoperative Frankel neurological function classification was grade B in 7 cases, grade C in 13, grade D in 18, and grade E in 10. Among them, 36 patients had lesions involving two vertebrae (8 cases of cervical vertebrae 3–4, 12 cases of cervical vertebrae 4–5, 6 cases of cervical vertebrae 5–6, and 10 cases of cervical vertebrae 6–7) and 12 patients had lesions involving more than three vertebrae (four cases of cervical vertebrae 3–5, four cases of cervical vertebrae 4–6, and four cases of cervical vertebrae 5–7). All the patients had different degrees of kyphosis, and the Cobb angle of kyphosis was 22.86 ± 2.15°. Preoperative ESR was 41.4 ± 7.2 mm/h, and CRP level was 44.3 ± 4.6 mg/L.

### Perioperative results

The patients were followed up for 13–43 months, with an average of 21.46 ± 1.52 months. The operation time was 102–185 min, with an average of 120. 4 ± 21.2 min. The mean intraoperative blood loss was 82.3 ± 18.2 mL, with a range between 55 and 190 mL. The mean time of hospitalization was 12.25 ± 2.15 days, with a range between 10 and 22 days. Functional exercise could be performed under the protection of the neck brace 2–4 days after the operation.

### Postoperative results

**Fig. 2:**
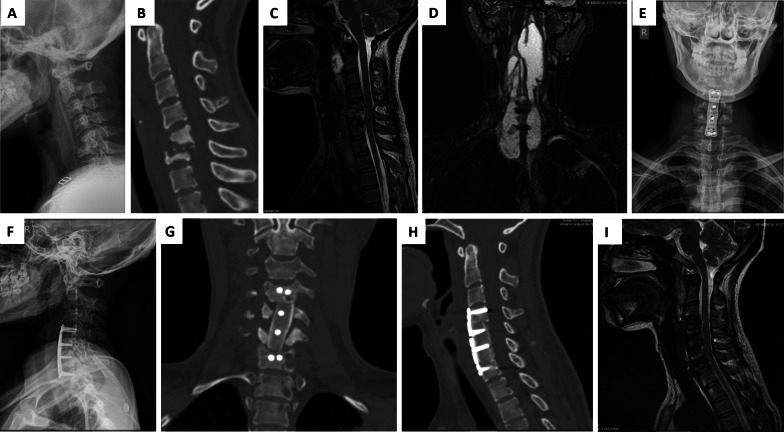
A 39-year-old female patient with cervical tuberculosis. **A** Preoperative X-ray showing bone destruction in the lower cervical spine and narrowing of the intervertebral space. **B** Preoperative computed tomography (CT) showing bone destruction, narrowing of the intervertebral space, marked swelling of the prevertebral soft tissue, and formation of dead bone and abscesses. **C**, **D** Magnetic resonance imaging (MRI) showing vertebral bone destruction and intraspinal mass leading to significant compression and thinning of the cervical spinal cord. **E**–**I** Postoperative X-ray, CT, and MRI showing no loosening or fracture of screws, the implanted iliac crest has fused, the physical curvature of the cervical spine has been restored, and cervical cord compression has been released

At the last follow-up, the Cobb angle of lower cervical kyphosis, neck VAS score, ESR and CRP levels were significantly lower than those before surgery, and the differences were statistically significant (Table 1). The clinical symptoms of the patients improved significantly after surgery. At the last follow-up, 8 cases recovered to grade D, and 40 recovered to grade E (Table 2). The bone graft was completely fused in all patients, and the bone fusion time was 3–6 months, with an average of 4.16 ± 0.47 months. A typical patient is shown in Fig. 2.

**Table 1:** Comparison of preoperative and postoperative indicators at the last follow-up

Item	Cobb angle ($\overline{x}$ ± s, °)	VAS score ($\overline{x}$ ± s)	ESR ($\overline{x}$ ± s, mm/h)	CRP ($\overline{x}$ ± s, mg/L)
Preoperative	22.86 ± 2.15	6.3 ± 0.25	41.4 ± 7.2	44.3 ± 4.6
Last follow-up	4.63 ± 0.24	1.5 ± 0.05	7.3 ± 1.2	4.6 ± 0.3
*P*-value	*P* < 0.05	*P* < 0.05	*P* < 0.05	*P* < 0.05

**Table 2:** Status of different Frankel grades

Grades	Preoperative (*n*)	Postoperative third month (*n*)	Last follow-up (*n*)
B	7	1	0
C	13	6	0
D	18	14	8
E	10	27	40

### Postoperative complications

All surgical incisions healed in one stage without infection or sinus formation. None of the patients had loosening, detachment, or rupture of the internal fixation, and no recurrence occurred. Hoarseness occurred in three patients (6.25%, 3/48) after the operation, and the symptoms disappeared after nutritional nerve treatment. Mild pain in the iliac crest area was left in eight patients (16.67%, 8/48), and the pain in the iliac crest area disappeared in six patients at the last follow-up.

## Discussion

Cervical tuberculosis lesions are mainly in the cervical space and invade the adjacent vertebral bodies at the same time, causing cervical instability and kyphosis. The diseased tissue invades the spinal canal backward, compresses the spinal cord, and causes dysfunction [9]. Abscesses can break through the anterior longitudinal ligament and accumulate behind the longus cervix muscle and its fascia, forming abscesses in the throat wall and posterior esophagus [10]. The objective of surgical treatment of cervical tuberculosis is to remove the lesion, restore spinal cord function, correct cervical kyphosis, reconstruct the physiological curvature, and maintain stability [11].

Because Mycobacterium tuberculosis mainly invades the anterior and central columns of the cervical spine, and cervical spinal cord compression comes from the ventral side of the spinal cord, anterior debridement, spinal cord decompression, correction of kyphosis, and cervical stability reconstruction are the main surgical methods [12]. Wang et al. proposed a treatment protocol of Xiangya Institutes of Medical Sciences cervical tuberculosis grading system: conservative treatment for grade 1, anterior debridement and fusion for grade 2, and a combined anterior–posterior approach for grade 3 lesions) [8]. The anterior approach allows direct access to the lesion and relatively complete removal of the abscess, dead bone, and granulation tissue [13]. Anterior surgical methods include simple debridement, debridement and bone grafting, anterior one-stage debridement and bone grafting, and anterior internal fixation. In most cases of spinal tuberculosis with indications for surgery, anterior debridement and bone graft fusion can be performed without involvement of the posterior adnexa [14]. Anterior internal fixation is more effective than posterior internal fixation in correcting deformity and reconstructing stability, and can be considered in all cases without special circumstances [15]. In our opinion, thorough debridement of the lesion involves removal of all necrotic tissue, pus, caseous matter, dead bone, granulation tissue, necrotic intervertebral discs in the lesion area, and preservation of healthy and subhealthy tissues. Extensive resection, especially of the entire vertebral body, should be avoided because it aggravates trauma and bone defects, which is not conducive to the repair of tuberculosis lesions. Some scholars also believe that when the scope of tuberculosis transformation exceeds two vertebral bodies and the scope of anterior reconstruction crosses three vertebral spaces, it is recommended to adopt phased posterior and anterior combined surgery, with posterior fixation decompression in the first stage and anterior decompression and reconstruction fixation in the second stage [16]. The purpose of posterior surgery is to enhance the stability of the cervical spine and restore the curvature of the cervical spine through the posterior nail rod system. However, the nutritional status of tuberculosis patients is poor, and the greater trauma of combined anterior and posterior surgery will further increase the difficulty and risk of surgery, and the choice should be careful. In this group of patients, anterior debridement was performed, which could directly reach the lesion. The abscess, dead bone, caseous tissue, and granulation tissue were removed under direct vision, and spinal cord compression was completely relieved. Therefore, neurological function of the patients improved to varying degrees.

Complications of anterior cervical surgery include surgical approach-related complications, decompression-related complications, bone graft fusion-related complications, and internal fixation-related complications. However, laryngeal nerve and recurrent laryngeal nerve injury are the most common complications, with an incidence of 2.18% and 0.97%, respectively, and aggravating spinal cord and nerve damage is the most serious complication of anterior cervical surgery [17, 18]. There are important anatomical structures, such as nerves, blood vessels, trachea, and esophagus, in front of the cervical spine. Long-term excessive traction during the operation will lead to early esophageal injury, and long-term friction between the anterior plate and esophagus will also lead to delayed esophageal perforation [19]. In our experience, loosening the retractor or adjusting the position of the retractor after 0.5 h of intraoperative esophageal traction can avoid esophageal perforation caused by prolonged esophageal traction. In this study, hoarseness occurred in three patients (6.25%, 3/48) after the operation, and the symptoms disappeared after nutritional nerve treatment. Mild pain in the iliac crest area was left in eight patients (16.67%, 8/48), and the pain in the iliac crest area disappeared in six patients at the last follow-up. These may be related to the longer operation time, larger operation scope, and need to take a large iliac crest during the operation. For such patients, a more elaborate operation is required to reduce the incidence of these complications. In conclusion, careful preoperative planning, comprehensive perioperative management, and skilled surgical techniques are considered the most important factors for reducing complications and residual symptoms.

At present, research on anterior surgery for cervical tuberculosis has shown that the operation has achieved different degrees of short-term or long-term efficacy, and the postoperative deterioration or recurrence in patients is mostly caused by the insufficient time of chemotherapy and the failure to control the lesion [20, 21]. Therefore, regular postoperative reexamination of liver and kidney function, ESR and X-ray, regular full course of antituberculosis drugs, full understanding of lesion healing and stability, and strict postoperative chemotherapy can ensure the effect of surgery. In this study, the patients were treated with antituberculous drugs strictly before and after surgery and achieved good clinical efficacy at the last follow-up. In our opinion, the prognosis of patients with cervical tuberculosis is not only related to surgical management, but more importantly, regular antituberculosis drug treatment in the early, full, combined, and appropriate principles.

The use of implants is very important in cervical tuberculosis surgery, and their main role is to achieve osseous fusion of the lesion site to provide structural support [22]. The effect of bone graft fusion directly affects the effect of surgery; therefore, whether the choice of implant is reasonable directly affects whether the bone graft fusion can achieve the ideal state. The most commonly used implant in clinical practice is the autogenous iliac bone, which is generally a three-sided cortical iliac bone block that has a good three-dimensional support effect and is conducive to improving the fusion rate [23]. Raja et al. performed anterior cervical decompression and autologous iliac bone graft fusion in 44 patients with cervical tuberculosis. All patients had good neurological function after surgery, and no postoperative complications were found [24]. However, the autogenous iliac crest also has some drawbacks, such as pain and infection at the site of bone retrieval and the risk of neurovascular and urethral injury. Injury to the pelvis can also lead to pelvic fractures and instability. In view of the above problems, Zhang et al. attempted to use a vascularized autologous fibula as an implant in the treatment of a patient with multilevel cervical tuberculosis. The cervical spine function of the patient recovered well after surgery, and the fibula as an implant has more advantages in terms of length [25]. Therefore, this method may provide a new treatment strategy for long-segment cervical tuberculosis. Pain after autogenous iliac crest removal does not affect its status in bone graft fusion, and the autogenous iliac crest remains the gold standard for endografts in cervical tuberculosis surgery. Proper treatment of bone extraction sites, mature bone extraction techniques, and the development of minimally invasive surgery have improved the effect of bone extraction and reduced the incidence of postoperative pain. The cervical spine is characterized by abundant blood supply, high drug concentration, and abundant soft tissue, and the autogenous iliac bone graft has the advantages of good bone conductivity, inductance, and osteogenic activity [26]. The results of this study indicate that autogenous iliac bone can be safely used for structural support therapy after removal of cervical tuberculosis lesion under effective antituberculosis therapy. Short segmental fixation preserves the uninvolved bone parts above and below the affected vertebra to insert the internal fixation, thereby preserving the adjacent healthy intervertebral space and vertebral body, minimizing the injury of cervical tuberculosis lesion removal, and facilitating healing and bone fusion [13].

In recent years, the advantages of titanium as a bone graft fusion material have also become increasingly prominent, with good effects on restoring the physiological curvature of the cervical spine, rebuilding the stability of the cervical spine, promoting bone graft fusion, and improving the cure rate of cervical tuberculosis [27]. However, the subsidence phenomenon of titanium cages after implantation is worth further study [28]. A recent study investigated the effectiveness of allograft bones combined with poly-ether-ether-ketone (PEEK) cages or titanium mesh cages (TMCs) in the management of cervical spinal tuberculosis, and they found that allograft bone combined with PEEK cages and TMCs could bring about favorable clinical results in patients with cervical spinal tuberculosis. This method could be an alternative to autologous bone grafting method in the management of certain cases [29]. In recent years, many new technologies have emerged, for example, three-dimensional printing-assisted cervical anterior bilateral pedicle screw fixation of artificial vertebral body for cervical tuberculosis [30].

This study had some limitations. First, this was a retrospective study, which inevitably resulted in selection bias. Second, the overall postoperative follow-up time was short, which may have underestimated the incidence of mechanical complications, such as aseptic loosening and screw fracture. Third, this was a small-sample study, and a large-scale study in multiple centers is needed. Finally, the effect on bone growth needs to be confirmed with a longer follow-up period.

## Conclusion

For patients with lower cervical tuberculosis, based on early diagnosis and antituberculosis drug treatment, anterior debridement combined with autogenous iliac bone graft fusion and internal fixation can completely remove tuberculosis foci, relieve spinal cord compression, rebuild cervical stability, and obtain good clinical efficacy.

## Data Availability

The raw data supporting the conclusions of this article will be made available by the authors without undue reservation.
